# Medico-Economic Evaluation of a Telehealth Platform for Elective Outpatient Surgeries: Randomized Controlled Trial

**DOI:** 10.2196/76730

**Published:** 2025-08-26

**Authors:** Florian Robin, Maxim Roy, Alexandre Kuftedjian, Marie-Eve Desrosiers, Frederic Lavoie, Marie-Pascale Pomey, Alexandre Castonguay, David Benatia, Guy Paré

**Affiliations:** 1 Department of Anesthesiology and Pain Medicine, Université de Montréal, Pavillon Roger-Gaudry, local S-712, 2900, boul. Édouard-Montpetit, Montreal, QC, H2X 0C1, Canada, 1 514 343-6466; 2 Department of Anesthesiology, Centre Hospitalier de l’Université de Montréal, Montreal, QC, Canada; 3 Research Centre, Centre hospitalier de l'Université de Montréal, Montreal, QC, Canada; 4 Department of Anesthesiology, Centre Hospitalier de l’Université de Montréal, Montreal, QC, Canada; 5 Centre Hospitalier de l’Université de Montréal, Université de Montréal, Montreal, QC, Canada; 6 School of Public Health, Université de Montréal, Montreal, QC, Canada; 7 Faculty of Nursing, Université de Montréal, Montreal, QC, Canada; 8 Department of Applied Economics, HEC Montréal, Montreal, QC, Canada; 9 Research Chair in Digital Health, HEC Montréal, Montreal, QC, Canada

**Keywords:** ambulatory surgery, postoperative follow-up, telemedicine platform, cost-effectiveness, cost-utility, perioperative medicine

## Abstract

**Background:**

The increasing prevalence of ambulatory surgeries has highlighted the need for effective postoperative follow-up. While telemedicine represents a promising option for perioperative support and postoperative monitoring, evidence of its actual benefits remains limited.

**Objective:**

This study aims to evaluate the medico-economic impact of a personalized telemedicine platform for postoperative follow-up in day-surgery patients in terms of cost-effectiveness and cost-utility.

**Methods:**

This single-blinded, 2-group randomized controlled trial was conducted at the Centre Hospitalier de l’Université de Montréal (CHUM) from August 2022 to September 2023. Adults undergoing elective day surgery were randomized into 2 groups: the intervention group, which received postoperative follow-up via the LeoMed telemedicine platform, and the control group, which received standard care. The intervention group used a personalized telehealth platform offering preoperative education, psychological support, and postoperative monitoring through daily follow-up forms sent to patients’ smartphones. Alerts generated by patient responses were reviewed by CHUM’s telehealth support unit. The primary outcome was unanticipated health care usage, including emergency visits, readmissions, and medical consultations within 30 days postprocedure. Secondary outcomes included gained quality-adjusted life years (QALYs), patient satisfaction, health care costs, and greenhouse gas emissions. Demographic and outcome data were summarized using descriptive statistics; categorical variables were reported as frequencies and percentages, and continuous variables as means with standard deviations. Between-group comparisons were conducted using appropriate statistical tests by the HEC Montréal health economics team, following an intention-to-treat approach.

**Results:**

Of 1411 patients screened, 1214 were randomized, with 436 in the intervention group and 445 in the control group analyzed. Compliance with the platform was high, with a mean compliance index of 0.89 in the intervention group. No significant differences in unanticipated health care usage were observed. The average cost of unplanned care was CAD $370 (US $275) in the control group versus CAD $323 (US $239) in the intervention group (*P*=.60). The intervention group demonstrated a statistically significant QALY gain at postoperative day 14 (0.002; *P*=.01), but the difference was no longer significant at day 30 (0.001; *P*=0.14). There were also no significant differences in GHG emissions between the groups, with the intervention group emitting an average of 0.870 kg CO₂-eq compared with 1.055 kg CO₂-eq in the control group (*P*=.52). However, patient satisfaction was significantly higher in the intervention group at both days 14 (*P*=.02) and 30 (*P*<.001).

**Conclusions:**

This trial demonstrates the potential of telemedicine platforms to enhance postoperative care in ambulatory surgery settings. While no significant reductions in health care usage were observed, the intervention improved QALYs and patient satisfaction, suggesting potential cost-utility benefits. Larger trials are needed to confirm these findings and explore the impact on long-term recovery and health care savings.

## Introduction

Perioperative care pathways have undergone profound changes over the past decade. The development of Enhanced Recovery After Surgery (ERAS) programs and minimally invasive surgical techniques has significantly reduced hospital stays and promoted ambulatory procedures for a broader range of surgeries [1]. In Canada, more than 2.3 million ambulatory procedures are performed annually as of 2024 [2]. These procedures are no longer restricted to ASA 1‐2 (American Society of Anesthesiologists Physical Status Classification System) patients or low-risk, short-duration surgeries [3]. However, the rate of postoperative complications and the level of postoperative pain are not significantly lower in outpatient surgeries compared with inpatient procedures [4].

A decade ago, patients benefited from daily morning check-ins by health care professionals to monitor vital signs, detect complications, and provide pain management support in the days following surgery. Today, patients are expected to take on much of their recovery independently. Consequently, many events may be underreported to health care professionals and, as a result, underestimated by health care institutions. For instance, postoperative opioid use remains a significant issue, with recent studies showing that nearly 10% of patients may still be using opioids 3 months after an outpatient procedure [5]. This highlights that the health care system has not adapted sufficiently to provide optimal perioperative support and postoperative follow-up.

Medical associations have advocated for establishing contact between the patient’s discharge and the first few postoperative days. For example, the Haute Autorité de Santé (HAS) in France mandates that health care facilities contact patients within 1-3 days after surgery [6]. However, this timeframe is based on arbitrary decisions rather than robust scientific evidence, and not all patients necessarily require postoperative follow-up. Several authors, such as Dahlberg et al [7], have proposed a patient-centered approach where patients determine if and when follow-up contact is needed.

New technologies offer significant potential to enhance remote follow-up methods. However, for these postoperative monitoring solutions to be widely adopted in routine practice, their clinical and economic benefits must first be demonstrated. To date, only a limited number of studies in the literature address this critical issue [8].

In 2022, the Department of Anesthesiology at the Centre Hospitalier de l’Université de Montréal (CHUM) introduced an automated, personalized perioperative platform integrated with its telehealth service. This advanced digital health solution is smartphone-accessible, offering perioperative support and remote postoperative follow-up [9]. Designed according to a patient-centered care model, the platform offers structured monitoring while allowing patients to engage at their own pace based on perceived recovery needs, thereby reinforcing autonomy in the digital perioperative setting. Unlike conventional systems, LeoMed integrates automated alerts, personalized educational content, and interactive features to deliver a more adaptive and patient-driven follow-up experience.

We hypothesize that implementing this telehealth platform at CHUM will (1) reduce the frequency of emergency department visits, unplanned medical consultations, unnecessary calls to Provincial Health Information Line, and unplanned hospital readmissions by offering more tailored health support, and (2) improve the quality of life 30 days after surgery through timely and effective recognition of postoperative complications.

The primary objective of this study is to evaluate the medico-economic impact of incorporating this patient-tailored telecare platform into perioperative pathways for day-surgery patients, through a cost-effectiveness and cost-utility analysis.

## Methods

### Trial Design

This single-center, single-blind, 2-group randomized controlled trial was conducted at CHUM between August 2022 and September 2023. Patients were divided into 2 groups: one group received perioperative support through a personalized telehealth platform (LeoMed), while the other group received standard care. The study protocol was published in April 2023 [9].

### Study Population

All patients over the age of 18 undergoing elective day surgery at CHUM were eligible. The inclusion criteria required patients to have internet access and be able to understand both written and spoken communication in either French or English. Patients were excluded at two stages: (1) before randomization, if they did not meet the inclusion criteria or chose not to participate, or if surgeries were postponed, and (2) after randomization, if patients withdrew their consent, met exclusion criteria not identified during the telephonic initial approach, were hospitalized on the day of surgery, or if surgeries were postponed at the last minute.

**Figure 1.**
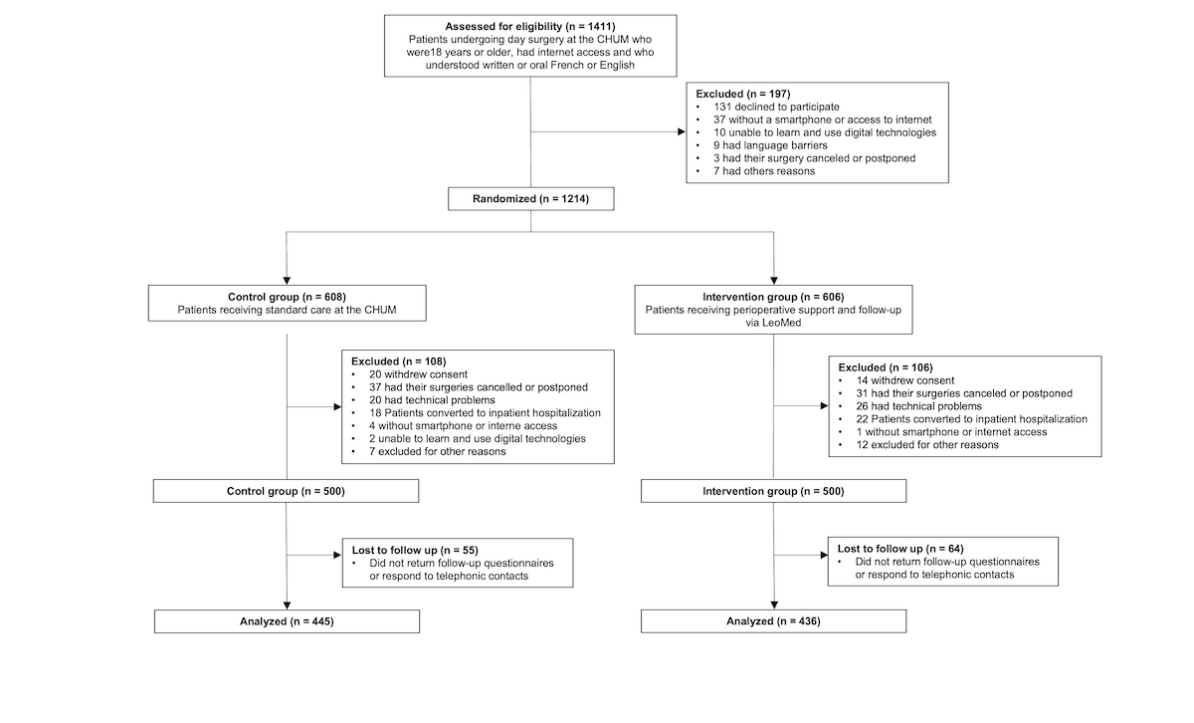
Flow chart. CHUM: Centre Hospitalier de l’Université de Montréal;

### Randomization and Blinding

Patients were initially approached by phone 2 days before their surgery, based on the operating schedule. To manage the resources available in the research unit, we limited the number of patients included each day to a maximum of 5. During the assessment, a research assistant verified the inclusion criteria and provided verbal information about the study. If the patient agreed to participate, they could provide written consent either electronically via email or give verbal consent and sign in person on the morning of their surgery upon arrival at the day surgery unit. All enrolled patients were electronically randomized in a balanced 1:1 ratio into either the intervention group or the control group, using blocks of 2-4. The randomization list was created with the MedSharing Randomizer for Clinical Trials web application. Patients were not informed of their randomization group; however, due to the nature of the intervention, they were not blinded to their group allocation.

### Intervention

The intervention consisted of perioperative support through a personalized telehealth platform (LeoMed Technologies Inc). Specifically, patients in the intervention group received a link the day before their surgery, prompting them to access the platform to prepare for their procedure. Preoperatively, the platform directed patients to CHUM’s digital portal, which centralized health sheets outlining the perioperative process. In addition, customized psychological support videos, developed in collaboration with CHUM’s communications team, were made available to address patient concerns and provide comprehensive guidance. Postoperatively, the platform sent electronic follow-up forms to participants’ phones daily during the first 4 days and again on the seventh day, prompting them to evaluate their health status. These forms included questions about potential surgical site infections and symptoms such as redness, pus, fever, and wound pain. Patient responses were analyzed automatically by an artificial intelligence algorithm, generating alerts visible to CHUM’s telehealth service team. This service, staffed by approximately 50 specialized nurses trained in remote health care delivery, provides remote support and coordinates interventions with CHUM’s medical and paramedical teams. As part of this project, nurses contacted patients within a maximum of 8 hours following an alert to ensure appropriate care was delivered.

Participants assigned to the control group received the hospital’s standard care, which included a preoperative phone call from a scheduling agent on the day before surgery. Postoperative follow-up for control group patients depended on the surgical specialty, with the timing of follow-up visits varying by surgeon and type of surgery, typically ranging from a few days to several weeks. However, like patients in the intervention group, control group patients were required to log in to the platform the day before surgery. At that time, they completed the preoperative quality of life assessment score (EQ-5D-5L VAS), as in the intervention group.

In both groups, the platform sent follow-up evaluation forms on postoperative days (POD) 14 and 30 to assess variables needed for the medico-economic analyses. These included the postoperative EQ-5D-5L VAS score, health care usage, and patient satisfaction.

### Compliance

To ensure compliance in the intervention group, we developed a Compliance Index to assess patient engagement with the telehealth platform during the immediate postoperative period, specifically the first 4 days. Compliance was measured by calculating the frequency of questionnaire responses, which served as a proxy for engagement. A participant who responded to all 4 questions received a compliance score of 1, while a participant who did not respond to any received a score of 0.

### Data Collection

Demographic data (age, gender, type of surgery, and type of anesthesia) were extracted from CHUM’s electronic medical record (EMR) system (Oacis; Telus Health). Economic and health-related variables were collected from the telehealth platform via electronic questionnaires completed by patients preoperatively and on POD 14 and 30. To ensure the accuracy of the information provided, all patients who reported complications or events, such as any health care usage, were systematically contacted by our research assistants via phone. Patients who did not complete the electronic questionnaires also received a reminder call. For each patient, the data were systematically recorded and securely stored on a local server before being extracted into 2 Excel (Microsoft) databases. These data were cross-referenced with those extracted from the EMR.

### Outcomes

#### Cost-Effectiveness

The cost-effectiveness analysis considered costs associated with unanticipated health care usage, defined as calls to the CHUM local health information line, calls to the Quebec health information line (811), emergency department visits, and unplanned readmissions or medical visits (to family doctors or outpatient clinics) related to the procedure within the first 30 days postprocedure. Table 1 presents the average unit costs of health care services and the sources used for estimating the categories of health care costs. We computed an additional cost variable related to additional health care usage. The variable “Costs+Transportation” adds the round-trip transportation costs from the patient’s postal code to the respective service (nearest emergency department, specified clinic, or doctor). These transportation costs are based on our estimates of travel distance and time, as reported in the “Other Outcomes” section.

**Table 1.** Categories of health care costs.

Cost categories	Mean cost (CAD $; US $)	Sources
Family doctor or walk-in clinic visit	54.0; 40	Billing reference guide (2024). Santé Inc. Code 15823.
Emergency room visit (without additional tests)	1435.0; 1062	Tariffs for hospital visits to the CHUM^a^, MSSS^b^ (2023)
Hospitalization (per day, all-inclusive)	8286.0; 6132	Tariffs for hospital visits to the CHUM^a^, MSSS^b^ (2023)
Provincial 811 help line (per call)	46.0; 34	Annual financial reports of institutions 2022‐2023 - MSSS.
Health line (CHUM; per call)	103.0; 76	Internal data from CHUM 2023‐2024 (AS-471 ca 6240)
Telehealth platform (LeoMed) access (per patient)	3.7; 2.8	Internal CHUM estimates for telehealth platform usage (2023‐2024)^c^

a CHUM: Centre Hospitalier de l’Université de Montréal.

b MSSS: The Ministry of Health and Social Services.

c Technology costs were calculated based on prevailing license prices during the study period and amortized on a straight-line basis over 5 years.

#### Cost-Utility

The cost-utility analysis evaluates the gained quality-adjusted life years (QALYs). The EQ-5D-5L visual analog scale (VAS) was selected as the instrument for assessing quality of life and deriving utility values for health technology appraisals [10]. This choice was made based on the recommendations from the latest update to the economic evaluation guidelines published by the French National Authority for Health [11]. The EQ-5D-5L comprises 2 components: an assessment of health status across 5 dimensions—mobility, self-care, usual activities, pain or discomfort, and anxiety or depression—and a visual analog scale (EQ VAS) that measures the patient’s overall perception of health. This tool was used to evaluate health outcomes on POD 14 and 30, and the results were compared with preoperative baseline scores.

#### Other Outcomes

Greenhouse gas (GHG) emissions associated with the additional health care use were also evaluated. To calculate GHG emissions, we estimated the likely travel time from the patient’s residence to the designated medical facility, assuming a typical weekday morning departure scenario (Wednesday, 15 May 2024, 8:00 AM, Montreal Time). The geographical coordinates of hospitals, emergency rooms, or other medical facilities visited by participants were determined using a comprehensive geocoding process implemented in the R programming environment (R Foundation for Statistical Computing). This process employed the Googleway package to perform geocoding and calculate distances and travel times in driving mode, based on explicit and implicit address data provided in the dataset. Finally, patient satisfaction with care services was assessed in both groups using Likert scales adapted from Sicotte et al [12].

### Statistical Analysis

Data analysis was conducted using R. Demographic data were summarized using descriptive statistics. Categorical data were presented as frequencies and percentages, while continuous variables were reported as means with standard deviations. Health care service usage was analyzed alongside associated costs and carbon dioxide (CO₂) emissions. All financial calculations were expressed in Canadian dollars. QALYs were calculated using EQ-5D-5L scores converted into utility values based on the Canadian Time Trade-off Value Set [10]. Utility scores were derived using a predefined function and adjusted for the number of days since surgery (14 or 30 days) divided by 365. This method accounts for differences across all EQ-5D-5L dimensions. Our analysis primarily followed a modified intention-to-treat (mITT) principle, whereby all randomized participants were included except those with completely missing outcome data for a given analysis. No imputation was performed, and participants with incomplete medico-economic data were excluded from the corresponding analyses. To detect differences between the 2 groups, as well as gender differences, we used the chi-square test, Mann-Whitney *U* test, or independent Student *t* test, as appropriate. This study adhered to the SPIRIT (Standard Protocol Items: Recommendations for Interventional Trials) 2013 Statement [13] and the CHEERS (Consolidated Health Economic Evaluation Reporting Standards) guidelines [14] (the CONSORT [Consolidated Standards of Reporting Trials] checklist is provided in Checklist 1).

### Sample Size

Our sample size calculations were based on the study of Jaensson et al [15], who suggested that better postoperative recovery—indicated by a reduction in symptoms and complications—would likely result in fewer postoperative calls to health information lines, visits to the emergency department, unplanned readmissions, and associated medical visits, thus lowering overall costs [15]. In their study, they found that patients in the intervention group, who used a recovery assessment mobile app, exhibited better recovery outcomes (as measured by the global Swedish Web Version of the Quality of Recovery [SwQoR] score) compared with those receiving standard care on POD 7 (effect size 0.21; *P*<.001).

Using a significance level of .05 and a statistical power of .90, the necessary group size was calculated by G*Power (Heinrich Heine University) to require 357 participants per group. To accommodate a 20% loss to follow-up and potential protocol deviations, we planned to recruit 500 patients for each arm of the study, for a total of 1000 patients.

### Ethical Considerations

The study adhered to the principles of the Helsinki Declaration and was approved by the institutional review board and research ethics committee at CHUM (21.110). The trial was registered with ClinicalTrials.gov (NCT04948632).

## Results

### Overview

Between August 2022 and September 2023, 1411 patients were assessed for eligibility. After excluding 197 patients, 1214 participants were randomized into 2 groups: 608 in the control group and 606 in the intervention group (Figure 1). After randomization, 108 participants from the control group and 106 from the intervention group were excluded, primarily due to the following reasons: withdrawal of consent (n=34), surgery cancellations or postponements (n=68), technical problems (n=46), conversion to inpatient hospitalization (n=40), or other reasons (n=26). A total of 55 patients in the control group and 64 in the intervention group did not return follow-up questionnaires or respond to telephonic contacts, resulting in a loss to follow-up. Figure 1 illustrates the trial flowchart. Baseline characteristics are described in Table 2. Compliance was estimated as 0.89 in the intervention group.

**Table 2.** Patients' baseline characteristics.

Characteristic	Control group (n=500)	Intervention group (n=500)	*P* value
Age (years), mean (SD)	47.4 (13.1)	46.6 (12.9)	.29
Sex (female), n (%)	327 (65)	342 (68)	.31
Type of anesthesia^a^, n(%)			
General anesthesia	342 (70)	355 (72)	.39
Spinal anesthesia	40 (8)	39 (8)	.90
Peripheral nerve block	113 (24)	101 (21)	.25
Sedation	5 (1)	5 (1)	.90
Type of surgery, n (%)			
Orthopedics	111 (22)	96 (19)	.24
Plastic	100 (20)	106 (21)	.65
Gynecology	78 (16)	79 (16)	.94
Otolaryngology	60 (12)	63 (13)	.78
Oncology	61 (12)	60 (12)	.91
Hepatobiliary	14 (3)	16 (3)	.72
Thoracic	14 (3)	8 (2)	.20
Urology	11 (2)	15 (3)	.43
Others^b^	49 (<10)	56 (11)	N/A^d^
Distance and travel time, mean (SD)			
Distance from home to CHUM^c^ (km)	52.3 (14.2)	41.9 (11.1)	.06
Distance from home to closest ER^e^ (km)	12.8 (3.3)	13.0 (3.4)	.79
Travel time from home to CHUM (min)	47.8 (13.3)	39.1 (10.6)	.03
Travel time from Home to Closest ER (min)	18.3 (4.95)	18.9 (5.04)	.40

a Percentages do not add up to 100% for the type of anesthesia because of either the combination of peripheral nerve block and general anesthesia or conversion into general anesthesia.

b Neurosurgery, maxillofacial surgery, ophthalmology, vascular surgery, cardiac procedures, and radiology procedures, each comprising fewer than 10 patients.

c CHUM: Centre Hospitalier de l’Université de Montréal.

d N/A: not applicable.

e ER: emergency room.

### Primary Outcome

No significant differences were observed in unanticipated postoperative health care usage between the groups (Table 3). The mean cost of health care usage was CAD $370 (US $275) in the control group compared with CAD $323 (US $239) in the LeoMed group (*P*=.60). No significant difference was observed for the ‘Costs+Transportation’ variable (US $374 vs US $323; *P*=.33).

**Table 3.** Health care usage.

Use of health care services within 30 days following discharge	Control group, (N=445)		Intervention group, (N=436)		Odds ratio (95% CI)	*P* value
	Patients, n (%)	95% CI	Patients, n (%)	95% CI		
Family doctor or walk-in clinic visits	40 (8.99)	6.3-11.7	36 (8.26)	5.7-10.9	0.91 (0.57-1.46)	.54
Emergency room visit	37 (8.31)	5.711.0	30 (6.88)	4.5-9.6	0.81 (0.49-1.34)	.31
Hospitalization	10 (2.25)	0.9-3.7	7 (1.61)	0.5-3.0	0.71 (0.27-1.88)	.43
Provincial 811 help line	23 (5.17)	3.2-7.4	13 (2.98)	1.4-5.2	0.56 (0.28-1.13)	.18
Health line (CHUM^a^)	26 (5.84)	3.7-8.4	33 (7.57)	5.1-10.4	1.32 (0.78-2.25)	.06

a CHUM, Centre Hospitalier de l’Université de Montréal.

### Secondary Outcome

At POD 14, the intervention group demonstrated a statistically significant QALY gain of 0.002 compared with the control group (*P*=.01). Among the 5 dimensions of the EQ-5D-5L, autonomy, daily activities, and pain showed significant improvements in the intervention group at POD 14 (Table 4). At POD 30, the difference was no longer statistically significant (QALY gain=0.001; *P*=.14). No significant differences were observed between the groups for the EQ-5D-5L VAS scores at POD 14 and 30 (Table 4).

**Table 4.** Comparison of postoperative health scores.

Health variables	Control group (n=445)	Intervention group (n=436)	*P* value
Perceived Quality of Life (POD^a^ 14)			
EQ-5D-5L Global Score (VAS)^b^	7.4	7.7	.10
EQ-5D-5L dimensions			
Mobility	1.6	1.4	.11
Autonomy	1.6	1.4	<.001
Daily activities	2.1	1.8	<.001
Pain	2.3	2.0	<.001
Anxiety and depression	1.5	1.4	.08
Perceived Quality of Life (POD 30)			
EQ-5D-5L Global Score (VAS)	8.0	7.8	.10
EQ-5D-5L dimensions			
Mobility	1.4	1.3	.09
Autonomy	1.3	1.2	.09
Daily activities	1.7	1.5	.02
Pain	1.9	1.8	.02
Anxiety and depression	1.5	1.4	.12
Gained QALY^c^			
POD 14	0.000	0.002	.01
POD 30	0.000	0.001	.14

a POD: postoperative day.

b VAS: visual analog scale.

c QALY, quality-adjusted life years.

There were also no significant differences in GHG emissions between the groups, with the intervention group emitting an average of 0.870 kg CO₂-eq compared with 1.055 kg CO₂-eq in the control group (*P*=.52). However, patient satisfaction was significantly higher in the intervention group at both POD 14 and POD 30, with mean scores of 8.9 versus 8.4 (*P*=.02) and 8.8 versus 8.1 (*P*<.001), respectively.

## Discussion

### Principal Findings

This single-center, 2-group randomized controlled trial highlights the potential utility of telemedicine platforms in enhancing postoperative follow-up for ambulatory surgeries. While we observed no statistically significant reduction in unplanned health care usage, our study demonstrated a gain in QALYs at POD 14 in the intervention group. With willingness-to-pay thresholds for QALYs ranging from CAD $47,048.84 to CAD $73,936.87, the estimated economic benefit range from CAD $94 to CAD $148 per patient [16].

Several factors may explain these findings. Although the study did not demonstrate a significant difference in cost-effectiveness, we observed a trend toward reduced health care visits in the intervention group, similar to results reported by Temple-Oberle et al [17], who found no significant differences in unplanned health care contacts in a randomized trial evaluating a smartphone app for postoperative monitoring. This lack of significant results may reflect limited statistical power due to the sample size. Nevertheless, our findings contrast with previous studies that reported more favorable outcomes associated with app-based follow-up on health care usage. For instance, Armstrong et al [18] demonstrated a significant reduction in in-person visits following ambulatory breast reconstruction using a mobile app [18], while Lee et al [19] reported low rates of unplanned emergency department visits in a same-day discharge protocol for minimally invasive colorectal surgery supported by mobile health tools. Despite these differences, the observed QALY improvement at day 14 in our study suggests that patients in the intervention group may have experienced a faster subjective recovery during the initial postoperative period. At day 30, recovery levels were similar between groups, possibly reflecting natural convergence in postoperative recovery trajectories and a psychological adaptation to residual symptoms such as pain, fatigue, or reduced autonomy. While the QALY gain observed at postoperative day 14 (0.002) was statistically significant, its clinical interpretation remains modest. In the literature, the minimal clinically important difference (MCID) for QALYs varies widely depending on the population and condition. As emphasized by Revicki et al [20], the interpretation of QALY gains should consider the nature and duration of the intervention as well as the size of the target population. However, small utility gains may still be meaningful when considered at a population level, particularly in large-scale procedures such as ambulatory surgeries. In this context, even a modest per-patient gain could represent substantial cumulative benefit, especially given the approximately 2.3 million ambulatory procedures performed annually in Canada [2]. Future studies should aim to define MCID thresholds specific to short-term digital perioperative care interventions.

Despite the promise of telemedicine in postoperative care, few studies have demonstrated clear benefits of digital health solutions [21,22]. This is partly due to the heterogeneity of available platforms and the challenges of conducting large-scale multicenter trials with standardized solutions. Our study also showed higher patient satisfaction in the intervention group, corroborating existing literature suggesting that digital follow-up addresses a critical gap in postoperative care [23]. For instance, Day et al [24] demonstrated that automated messaging during the perioperative period enhanced satisfaction, improved health literacy, and fostered collaborative decision-making between patients and care teams. In addition, a meta-analysis of telemedicine interventions found that over 80% of patients receiving remote follow-up reported high satisfaction levels [25]. In line with these results, the high compliance index observed in our study (0.89) reflects strong patient engagement with the telehealth platform, suggesting both good implementation feasibility and user acceptability.

### Study Limitations

The single-center design of this trial limits the generalizability of findings to other settings, particularly those involving minor surgical procedures. However, this limitation underscores the broader challenges of implementing standardized telehealth platforms across multiple institutions. The observed trend toward reduced unplanned health care contacts and costs might have reached statistical significance with a larger sample size. In addition, the lack of participant blinding, inherent to the nature of the intervention, may have introduced response bias in subjective outcomes such as satisfaction and perceived recovery. This limitation is compounded by the use of self-reported measures which, although based on validated instruments, remain susceptible to perception and recall biases that could influence reported satisfaction or quality-of-life ratings. Furthermore, since all participants in the intervention group received access to the full LeoMed platform, we were unable to determine which specific components such as psychological support videos or educational modules contributed most to the observed benefits.

Moreover, health care costs may have been underestimated. For instance, hospitalization costs were calculated based on single-night stays and excluded extended emergency department visits that were not classified as formal admissions. Diagnostic testing costs associated with emergency visits were also not included.

Finally, we were unable to identify subgroups that might benefit more from the platform due to heterogeneity across age groups and surgical specialties. Although randomization was expected to balance baseline characteristics, some potential confounders such as previous experience with telehealth were not systematically collected and could not be adjusted for. Future research should focus on identifying high-risk populations that might derive the greatest benefit from telemedicine interventions.

### Conclusions

In conclusion, with the expansion of ambulatory surgery and the inclusion of more comorbid patients and increasingly complex procedures, ensuring effective follow-up care is becoming increasingly important. Our study demonstrated promising results regarding the cost-utility benefit of telemedicine platforms for perioperative monitoring. However, further large-scale, multicenter studies are needed to better determine the exact medico-economic impact and the clinical benefits for the broader patient population.

## Supplementary material

10.2196/76730 Checklist 1 CONSORT (Consolidated Standards of Reporting Trials) checklist.

## Abbreviations

ASA 1-2: American Society of Anesthesiologists Physical Status Classification System
CAD: Canadian dollars
CHEERS: Consolidated Health Economic Evaluation Reporting Standards
CHUM: Centre Hospitalier de l’Université de Montréal
CONSORT: Consolidated Standards of Reporting Trials
CO₂: carbon dioxide
EMR: electronic medical record
ERAS: Enhanced Recovery After Surgery
GHG: Greenhouse gas
HAS: Haute Autorité de Santé
MCID: minimal clinically important difference
mITT: modified intention-to-treat
POD: postoperative days
QALY: quality-adjusted life year
SPIRIT: Standard Protocol Items: Recommendations for Interventional Trials
SwQoR: Swedish Web Version of the Quality of Recovery
VAS: visual analog scale
